# Increasing gastric juice pH level prior to anti-*Helicobacter pylori* therapy may be beneficial to the healing of duodenal ulcers

**DOI:** 10.3892/etm.2013.880

**Published:** 2013-01-02

**Authors:** HONG-YUN FAN, JUAN WANG, GUO-CHAO YAN, XIAO-HUI HUO, LI-JUAN MU, JIAN-KUN CHU, WEI-WEI NIU, ZHI-YING DUAN, JIN-CHENG MA, JING WANG, ZHI-YU WANG

**Affiliations:** 1 Department of Gastroenterology, First Hospital of Hebei Medical University, Shijiazhuang, Hebei 050031;; 2 Department of Biotherapy, Fourth Hospital of Hebei Medical University, Shijiazhuang, Hebei 050011;; 3 Department of Laboratory, First Hospital of Hebei Medical University, Shijiazhuang, Hebei 050031;; 4 Endoscopy Centre, First Hospital of Hebei Medical University, Shijiazhuang, Hebei 050031, P.R. China

**Keywords:** *Helicobacter pylori*, *Helicobacter pylori* eradication, duodenal ulcer, gastric juice pH, omeprazole, clarithromycin

## Abstract

The aim of this study was to observe the efficacy of clarithromycin-based triple therapy for *Helicobacter pylori* (*Hp*)-infected duodenal ulcer when combined with different pH levels of gastric juices. A total of 160 patients with *Hp*-infected duodenal ulcers were randomly allocated into two groups. Patients in the treatment group (n=80) were administered a 20-mg dose of omeprazole twice daily for 1 week and then the treatment and control groups (n=80) received therapy for *Hp* infection and duodenal ulcers. We observed the ulcer healing stage, the content of anti-*Hp* IgA in gastric juice and the *Hp* eradication rate before and after proton pump inhibitor therapy in the two groups. Results revealed that the *Hp* eradication rate in the treatment group was 93% compared with 81% in the control group, and the difference was statistically significant (P<0.05). The ulcer healing rate in the treatment group was 93%, compared with 70% in the control group (P<0.05). A positive linear correlation was observed between gastric pH and the content of anti-*Hp* IgA in gastric juice (P<0.05). Increasing gastric pH prior to anti-*Hp* therapy may be beneficial to the eradication of *Hp* and for promoting the healing of duodenal ulcers.

## Introduction

Infection with *Helicobacter pylori* (*Hp*) is a substantial public health problem that affects 20–50% of the population in industrialized nations and up to 80% of the population in less developed countries (1). Furthermore, *Hp* is associated with a number of gastro-duodenal disorders (2–4), including gastric carcinoma, gastric mucosa-associated lymphoid tissue lymphoma and peptic ulcer disease, particularly duodenal ulcers, where this bacterium is observed in the stomachs of ∼95% of patients (5). It is widely recognized that the eradication of *Hp* accelerates duodenal ulcer healing and prevents ulcer relapse. However, recent studies (6) have indicated that increasing antibiotic resistance may be responsible for the current low *Hp* eradication rate by classical clarithromycin-based triple therapy.

Alternative approaches have attempted to increase the *Hp* eradication rate, including bismuth-containing quadruple therapy, non-bismuth-containing quadruple therapy, sequential therapy and levofloxacin-containing regimens (7). However, by changing the administration strategy of antibiotics to initially improve the environment in which they exert their effects, we may be able to enhance their *Hp* eradication efficacy.

Accumulating evidence suggests that overproduction of gastric acid is important in the development of ulcer disease. Although numerous factors are known to affect *Hp* eradication, gastric pH levels have attracted considerable attention. Our study aimed to observe the healing rate and *Hp* eradication rate of *Hp*-infected duodenal ulcers following clarithromycin-based triple therapy combined with different gastric juice pH levels. Furthermore, we aimed to explore new administration strategies which may elevate the *Hp* eradication rate by providing a more suitable environment for anti-*Hp* drugs.

## Materials and methods

### Ethics approval and consent

For this human clinical study, we obtained the approval of The Institutional Human Research Review Committee and the research carried out on humans was in compliance with the Helsinki Declaration. Verbal and written informed consent was obtained from each patient prior to study enrollment. Informed consent was composed of five parts: patient name, aim, expected benefits and possible risks of the study and the rights of the patient during the study.

### Case selection

We enrolled 160 cases of *Hp*-infected duodenal ulcers from the outpatients and inpatients of the First Hospital of Hebei Medical University, between February 2010 and October 2011. The inclusion criteria were as follows: i) Patients who were diagnosed with duodenal ulcers (active stage or healing stage) by gastroscopy; ii) patients who were *Hp*-positive, confirmed by a ^14^C-urea breath test and staining microscopy; and iii) patients with a treatment course of 4 weeks. The exclusion criteria were as follows: i) Patients who were under 18 years old; ii) patients whose ulcer was actively bleeding; iii) patients who were pregnant or lactating; iv) patients who were allergic to proton pump inhibitors (PPIs) or antibiotics; v) patients who had been treated with PPIs or relevant antibiotics up to 2 weeks ago; vi) patients with a history of alcohol or drug abuse; and vii) patients with heart, liver or kidney dysfunction. The suspension criteria were as follows: i) Patients exhibiting poor compliance and who were not able to take medicine according to the arrangement; ii) patients who experienced adverse reactions; and iii) patients who did not attend the follow-up or were lost to the study. The study was approved by the ethics committee of the Fourth Hospital of Hebei Medical University, Shijiazhuang, Hebei, China.

### Therapy methods

The patients (n=160) were randomly assigned into 2 groups (Table I). Eighty patients (44 males, 36 females) were allocated to the treatment group; the average age was 48.5±4.6 years (range, 21–68) and 56 cases were in A2 stage, compared with 24 in H1 stage. The remaining 80 patients (48 males, 32 females) were allocated to the control group, which had an average age of 47.6±4.5 years (range, 23–66) and 52 cases were in A2 stage, compared with 28 cases in H1 stage. Staging was performed as described previously (8). No statistical difference was identified between the two groups with regard to age, gender or ulcer degree. Prior to treatment, the gastric juice was collected by gastroscopy and stored at −20°C for pH testing and evaluation of anti-*Hp* IgA content in the two groups. Patients in the treatment group were initially administered a 20-mg dose of omeprazole enteric-coated capsules twice daily. After 1 week, gastric juice pH and anti-*Hp* IgA content were reexamined. For the following 1 week, a 0.5-g dose of clarithromycin disperse tablets twice daily and a 0.1-g dose of furazolidone twice daily were added. Omeprazole enteric-coated capsules were continued for a further 2 weeks. Two weeks after the course of treatment had ended, ^14^C-urea breath tests, gastroscopy and staining microscopy were carried out again and gastric juice pH and content of anti-*Hp* IgA were reexamined. The treatment course differed for the control group; a 20-mg dose of omeprazole enteric-coated capsules twice daily, a 0.5-g dose of clarithromycin disperse tablets twice daily and a 0.1-g dose of furazolidone twice daily were administrated for the first week. Antibiotics were subsequently stopped, while omeprazole continued for a further 3 weeks. Two weeks after this course of treatment had ended, ^14^C-urea breath tests, gastroscopy and staining microscopy were carried out again and gastric juice pH and content of anti-*Hp* IgA were reexamined.

### Treatment of biopsy specimens

The biopsy specimens were treated according to the method described previously (9). Biopsy specimens were collected from the duodenal bulb by endoscopy and placed into a transport medium [phosphate-buffered saline (PBS) at pH 7.2]. Within 3 h of the completion of endoscopy, the samples were placed onto Columbia Agar enriched with 5% hemolyzed horse blood. They were subsequently incubated at 37°C in a microaerophilic atmosphere (5% O_2_, 10% CO_2_ and 85% N_2_) for 5–6 days.

### Testing gastric juice pH

When the gastroscope reached the mucus lake in the fundus of the stomach, a thin plastic straw was used to collect gastric juice through the biopsy hole and gastric juice pH was tested with pH indicator paper.

### Preparation of antigen

The ultracentrifuged cell sonicate was prepared from a strain of *Hp, Hp* NCTC 11637, in accordance with the method proposed by Hirschl *et al*(10).

### Preparation of gastric juice for the enzyme-linked immunosorbent assay (ELISA) test

Gastric juice samples were prepared according to the method described by Rathbone *et al*(11). Samples were centrifuged at 2000 x g for 10 min, neutralized to pH 6.5–7 by 0.67 M Tris-HCI (pH 7.4) in 0.15 M saline and diluted to 1:100 for IgA detection.

### Evaluation of anti-Hp IgA levels in gastric juice

*Hp*-specific IgA antibodies were measured by ELISA, as described previously (12). The antigen preparation was diluted in a sodium carbonate-bicarbonate buffer (pH 9.6). Antigen (1 *μ*g/ml) was added to each well of the 12-well plates and these were incubated at 4°C for 18 h. The plates were washed three times with PBS solution containing 0.05% Tween-20, following which 2x100 *μ*l of diluted gastric juice was added to each well and incubated at 37°C for 1 h. After washing, 100 *μ*l peroxidase-labeled goat anti-human IgA antibody (Sigma, St. Louis, MO, USA) was added to each well and incubated at 37°C for 1 h. The plates were washed again and 100 *μ*l ortho-phenylenediamine dihydrochloride was added to each well as a substrate. The plates were incubated in the dark at room temperature for 30 min. The reaction was inhibited by adding 1M H_2_S0_4_ and the plates were read for optical density (OD) at 450 nm using a Dynatech MR5000 reader (Yokohama, Japan). Results were expressed as the mean OD ± SD. The results were interpreted as positive when the OD ratio was ≥1.0 for IgA antibodies.

### Diagnostic standard of Hp infection and criteria for Hp eradication

Patients who had a positive ^14^C-urea breath test and histological identification of *Hp* by biopsy (where *Hp* had been cultured from the biopsies or *Helicobacter*-like organisms were detected in microscopic slide biopsies, stained using the Gram method) concurrently were diagnosed as having a *Hp* infection. Two weeks after the end of the therapy, patients who had a negative ^14^C-urea breath test and tested negative for histological identification of *Hp* by biopsy were classed as having successful eradication of *Hp*. For the ^14^C-urea breath test, patients were administered a ^14^C urea capsule to swallow following an overnight fast. After 10 to 15 min, patients provided a single breath sample by blowing through a straw into a bottle of liquid; this was tested for radioactive CO_2_.

### Evaluation of ulcer healing

Ulcer healing refers to ulcer disappearance and is classified into 2 groups. S1 stage indicates that the red regenerating epithelium has completely covered the floor of the ulcer and the white coating has disappeared, thus it is also known as the ‘red scar’ stage. In S2 stage, the redness has returned to the color of the surrounding mucosa, thus it may also be referred to as the ‘white scar’ stage.

### Statistical analysis

SPSS 13.0 was used for statistical analysis and data referring to measurements are expressed in the form of average ± standard deviation. The Chi-square test was used to compare the two groups, where P<0.05 was considered to indicate a statistically significant difference.

## Results

### Gastric juice pH before and after Hp eradication

The gastric juice pH of the treatment group was significantly higher than that of the control group before *Hp* eradication (P<0.05), however there was no significant difference between the two groups after *Hp* eradication (P>0.05; Table II).

### Association of gastric pH with content of anti-Hp IgA in gastric juice before and after Hp eradication

**Figure 1.**
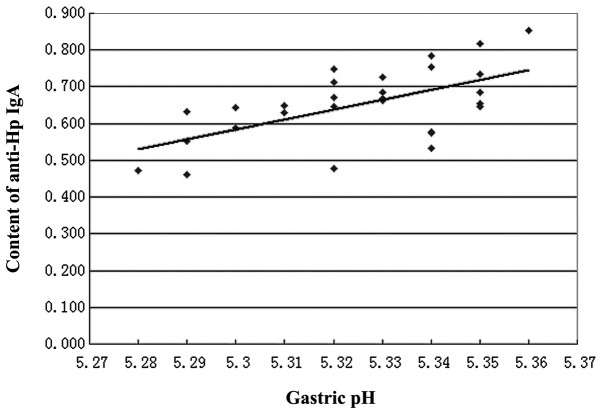
Correlation between gastric pH and content of anti-*Hp* IgA. A positive linear correlation was observed between gastric pH (x) and the content of anti-*Hp* IgA (y; r=0.5997, P<0.05, y=−13.661+2.6878x). *Hp*, *Helicobacter pylori*.

The content of anti-*Hp* IgA in gastric juice before *Hp* eradication was significantly higher in the treatment group than the control group (P<0.05). However, the content of anti-*Hp* IgA in gastric juice after *Hp* eradication was significantly lower in the treatment group than in the control group (P<0.05; Table III). In addition, a positive linear correlation was observed between gastric pH (x) and the content of anti-*Hp* IgA (y; r=0.5997, P<0.05, y=−13.661+2.6878x; Fig. 1).

### Efficacy of Hp eradication

All 80 patients in the treatment group completed their treatment, however 3 patients in the control group did not finish their treatment due to experiencing adverse reactions. The efficacy of *Hp* eradication was improved in the treatment group compared with the control group and this difference was statistically significant (P<0.05; Table IV).

### Ulcer healing rate

S2 stage achievement rate in the treatment group was significantly higher than in the control group (P<0.05; Table V).

### Adverse reaction

All 80 patients in the treatment group completed their treatment. Three patients in the control group did not finish their treatment due to experiencing nausea following ingestion of the anti-*Hp* drugs, however the remaining patients in this group did complete their treatment.

## Discussion

Since the discovery of *Hp* ∼30 years ago, our understanding of the pathophysiology of peptic ulcer disease has increased enormously. The presence of *Hp* leads to a sequence of pathophysiological events, including mucosal inflammation, impairment of the mucus-bicarbonate barrier, superficial epithelial cell damage, elevated serum gastrin levels with defective feedback control, a possible increase in parietal cell mass and gastric metaplasia in the duodenal cap. It has been well established that *Hp* is a critical contributor to the development of duodenal ulcers and that eradication of *Hp* accelerates duodenal ulcer healing and prevents ulcer relapse. Since the 1940s, it has been recognized that pH levels in the duodenal bulbs of patients with ulcer disease was lower than those without ulcers and that antacids or antisecretory therapy, which reduced the duodenal acid load, accelerated ulcer healing. Gradually, increasing numbers of studies started to explore the association between pH and *Hp* and this led to the conclusion that the duodenal acid load may be a critical factor in explaining the ability of *Hp* to colonize the duodenal bulb, via the precipitation of glycineconjugated bile salts. Furthermore, the combination of an elevated duodenal acid level and *Hp* infection is suggested to be a critical event in the pathogenesis of *Hp*-infected duodenal ulcers. It has also been reported that the minimum inhibitory concentration (MIC) of clarithromycin against *Hp* is an order of magnitude lower when pH is high (13). Therefore, among the numerous factors which are known to affect *Hp* eradication, gastric pH, which affects the activity of *Hp* and anti-*Hp* drugs, is one of the most crucial. Thus eliminating *Hp* in an environment with a suitable gastric pH may enhance *Hp* eradication, whilst also improving the healing quality of mucosa (4,14).

In this study, the treatment group was provided with acid-suppressing drugs for the first week, followed by anti-*Hp* therapy the week after. The control group received no treatment in the first week and the acid suppression and anti-*Hp* therapy were administered in the second week. Subsequently, the two groups were administered PPIs until the end of the therapy course. The main difference between the two groups was that gastric pH in the treatment group was changed by administering acid-suppressing therapy, thus the gastric juice pH levels were significantly higher than that of the control group before *Hp* eradication (P<0.05; Table II). This study observed the healing stage, the content of anti-*Hp* IgA in gastric juice and the *Hp* eradication rate in *Hp*-infected duodenal ulcers, after clarithromycin-based triple therapy in environments with different gastric pH levels.

*Hp* colonizes the microaerophilic environment in the protective layer of mucosal secretions, which are adjacent to the epithelium of the human stomach, particularly in the antrum. Numerous studies have shown that *Hp* is able to provoke a systemic and local immune response. Anti-whole *Hp* component specific IgA antibodies exist in the gastric juice, gastric tissue and saliva. Due to their ability to prevent the entrance of external foreign antigenic molecules, secretory IgA antibodies have generally been considered an immune barrier. However, secretory IgA (SIgA) antibodies have numerous functions, including the intracellular and serosal neutralization of antigens, activation of non-inflammatory pathways and homeostatic control of endogenous microbiota (15). Furthermore, studies have suggested that there is an association between the antibody titer of anti-*Hp* IgA and the intensity of inflammation and degree of *Hp* colonization in different parts of the stomach (16). This study has shown that *Hp*-specific IgA is correlated with gastric pH (Fig. 1). As indicated in Tables II and III and Fig. 1, higher gastric pH levels correspond with an increased content of anti-*Hp* IgA in gastric juice and vice versa. It is known that the optimum pH value of pepsin is 1.9, thus when the gastric pH is increased, its activity decreases and therefore the decomposition of anti-*Hp* IgA by pepsin is reduced. This leads to the increased content and enhanced immunocompetence of IgA, which causes an improvement in *Hp* eradication, as depicted in Table IV. As shown in Table III, the content of anti-*Hp* IgA in gastric juice after *Hp* eradication was significantly lower in the treatment group than in the control group (P<0.05). This may be explained by the higher *Hp* eradication rate in the treatment group and the short half-life of IgA. Additionally, the extent of the decrease in IgA content in the treatment group was larger than in the control group, which may reflect the difference in *Hp* eradication rate between the two groups.

Due to variation between *Hp* strains, their increasing resistance to antibiotics and numerous host and environmental factors, the eradiation of *Hp* is decreasing year by year (17,18). *Hp* colonizes the gastric epithelial surface and/or deeper into the mucus layer, where the median pH is 1.4. Due to the reduced activity of antibiotics in low pH levels and their decreased ability to penetrate the mucus layer, *Hp* elimination is unsuccessful. A reduction in pH negatively affects the activity of antibiotics, as demonstrated by a study which revealed a marked increase in the MIC of clarithromycin for clarithromycin-susceptible strains when pH was reduced; at pH 6.5, the MIC_50_ and MIC_90_ increased two- and eight-fold, respectively; at pH 5.9, eight- and 16-fold increases, respectively, were observed (13). Therefore, the negative effect of low gastric pH levels on the activity of anti-*Hp* drugs may be one of the most significant factors in the failure of *Hp* eradication. In the present study, we used the commonly administered clarithromycin-based triple therapy, which is a combination of omeprazole, clarithromycin and furazolidone. As has been illustrated in Tables IV and V, the *Hp* eradication rates and S2 stage achievement rates in the treatment group were significantly higher than those in the control group (P<0.05). The initial basic therapy with omeprazole in the treatment group relieved symptoms which were causing discomfort in patients, thus increasing their compliance for further treatment. More critically, it increased gastric pH, which had a positive effect on the efficacy of the antibiotics. Numerous studies have demonstrated that low pH conditions in gastric juice results in the rapid decomposition and decreased antibacterial efficacy of clarithromycin. Scholars have also investigated the impact of pH on the decomposition rate of clarithromycin, by calculating the decomposition rate constants of clarithromycin molecules in solutions and in human gastric juice. Such studies have demonstrated that the decomposition of clarithromycin in solutions and gastric juice proceeded in a pseudo-first order manner and the respective half-lives of clarithromycin were 0.1 and 1.3 h when the pH levels of the solutions were 1.0 and 2.0. Clarithromycin scarcely decomposed when the pH was above 5.0 (19). It has been suggested that the prolonged elevation of intragastric pH may increase the concentration of acid-labile antibiotics in gastric juice, prolong their effectiveness and improve the environment to allow the defense mechanisms of the host to exert their optimal effect. Thus the elevation of gastric pH may be a critical factor in *Hp* elimination and ulcer healing. *Hp* is also susceptible to furazolidone, which has a similar reaction to a change in gastric pH. In addition, PPIs have the direct ability to improve the content of anti-*Hp* IgA and partly eliminate *Hp*, which contributes to *Hp* eradication and ulcer healing. In summary, this synergistic effect may be due to the direct effect of omeprazole on the organism, the protection of antibiotics from acid suppression or a reinforcement in host defense mechanisms which accompany acid degradation.

Studies have revealed that the gastric pH levels of patients with duodenal ulcers were too low to allow the anti-*Hp* drugs to optimally exert their effects. Therefore, administering acid-suppressing therapy ahead of anti-*Hp* treatment may improve the gastric pH and compliance of patients, whilst also elevating the content of *Hp-*specific IgA and enhancing the sustainability, activity and efficacy of anti-*Hp* drugs, which is beneficial to the eradication of *Hp* and the healing of duodenal ulcers.

## Figures and Tables

**Table I.** Patient data.

		Age (years)			
Group	Number	Mean ± SD	Range	Gender (female/male)	Stage (A2/H1)
Treatment	80	48.5±4.6	21–68	36/44	56/24
Control	80	47.6±4.5	23–66	32/48	52/28

**Table II.** Gastric juice pH before and after eradication of *Hp* in the two groups.

	Gastric juice pH, mean ± SD	
Variable	Before *Hp* eradication	After *Hp* eradication
Treatment group	5.32±0.023	2.546±0.692
Control group	2.35±0.026	2.436±0.597
P-value	<0.05	>0.05

Hp, Helicobacter pylori.

**Table III.** Content of anti-*Hp* IgA in gastric juice before and after *Hp* eradication in the two groups.

	Content of anti-*Hp* IgA in gastric juice (mean OD ± SD)	
Variable	Before *Hp* eradication	After *Hp* eradication
Treatment group	0.658±0.175	0.173±0.013
Control group	0.456±0.198	0.202±0.039
P-value	<0.05	<0.05

*Hp, Helicobacter pylori;* OD, optical density.

**Table IV.** Comparison of *Hp* eradication rates between the two groups.

Variable	Effective cases	Ineffective cases	*Hp* eradication rate (%)
Treatment group	74	6	93
Control group^a^	62	15	81
P-value	-	-	<0.05

a Three patients in the control group did not finish treatment due to experiencing adverse reactions. *Hp*, *Helicobacter pylori*.

**Table V.** Comparison of S2 stage achievement rates between the two groups.

Variable	Effective cases^a^	Ineffective cases	S2 achievement rate (%)
Treatment group	74	6	93
Control group^b^	54	23	70
P-value	-	-	<0.05

a Effective cases refer to the patients who had achieved S2 stage.

b Three patients in the control group did not finish treatment due to experiencing adverse reactions.

## Abbreviations:

*Hp*: *Helicobacter pylori*;
PPI: proton pump inhibitor;
ELISA: enzyme-linked immunosorbent assay
